# Decreased Gray Matter Volume in the Frontal Cortex of Migraine Patients with Associated Functional Connectivity Alterations: A VBM and rs-FC Study

**DOI:** 10.1155/2022/2115956

**Published:** 2022-01-25

**Authors:** Zhijian Cao, Wenjing Yu, Zhengxiang Zhang, Maosheng Xu, Jiangnan Lin, Luping Zhang, Wenwen Song

**Affiliations:** ^1^Department of Radiology, The First Affiliated Hospital of Zhejiang Chinese Medical University, Hangzhou, China; ^2^The First School of Clinical Medicine, Zhejiang Chinese Medical University, Hangzhou, China; ^3^Department of Neurology, The First Affiliated Hospital of Zhejiang Chinese Medical University, Hangzhou, China; ^4^Department of Radiology, Hangzhou TCM Hospital of Zhejiang Chinese Medical University, Hangzhou, China

## Abstract

**Background:**

Resting-state functional MRI is widely used in migraine research. However, the pathophysiology and imaging markers specific for migraine pathologies are not well understood. In this study, we combined both structural and functional images to explore the concurrence and process of migraines.

**Methods:**

Thirty-four patients with a history of migraine without aura presenting during the interictal period (MwoA-DI), 10 patients with migraine without aura presenting during the acute attack (MwoA-DA), and 32 healthy controls (HCs) were recruited in this study. All participants underwent scanning via MRI. Voxel-based morphometry (VBM) and seed-based resting-state functional connectivity (rs-FC) analysis were used to detect the brain structural and associated brain functional connectivity.

**Results:**

In VBM analysis, a decrease of gray matter volume (GMV) in the middle frontal cortex was found in MwoA patients compared with HCs. The GMV of the middle frontal cortex had a negative correction with the duration of disease. In rs-FC analysis, the left middle frontal cortex (lower, VBM result) in both the MwoA-DA and the HC groups showed significantly increased functional connectivity with the left middle frontal cortex (upper) and left superior frontal cortex compared with MwoA-DI. The left middle frontal cortex (lower) in the MwoA-DI group also showed decreased functional connectivity in the left posterior cingulate cortex (PCC) compared with the HC group. The left middle frontal cortex (lower) in the MwoA-DA group demonstrated significantly increased functional connectivity in the left cerebellum lobule VI compared with the HC group.

**Conclusions:**

Our results demonstrated that the middle frontal cortex may serve as an important target in the frequency and severity of migraines due to its role in pain regulation through the default mode network, especially in the PCC. In addition, the cerebellum may modulate the pathophysiology of migraines by serving as a communication point between the cortex and the brainstem.

## 1. Introduction

Migraines are a very common cause for headaches. Yet, the pathophysiology of migraines is still not thoroughly understood [1]. Functional MRI is a noninvasive method widely used to reveal migraine pathophysiology. It can detect brain blood oxygen saturation and the activity of the brain functional area [2, 3]. Previous studies have found that the frontal lobe, occipital lobe, limbic system, default mode networks, sensorimotor cortex, and cerebellum play an important role in the development of migraines [4]. However, a study that reviewed 219 studies on migraine using rs-FC failed to pinpoint exact insights into its pathophysiology [5].

Brain structural changes can lead to functional changes. For example, chronic pain patients typically exhibit altered thickness and function of the cerebral cortex. Studies found that compared with the healthy controls, migraine patients have less gray matter volume (GMV) in visual areas. Another study also found patients with chronic migraine have thinner cortices in the bilateral insular cortex, caudal middle frontal gyrus, precentral gyrus, and parietal lobes [6, 7]. Anatomical colocalization of functional and structural changes raises the possibility that the observed changes may be the cause for the pain [8]. Combined analysis of both anatomical and functional alterations in migraine patients may be helpful in elucidating the underlying pathophysiological mechanisms of disease. Furthermore, several studies have only focused on migraines without aura presenting during the interictal period. A small body of studies have explored both the interictal period and the attack period in migraine patients.

In this study, we compared VBM and rsFC changes in patients with migraine without aura presenting during the acute attack (MwoA-DA) and migraine without aura presenting during the interictal period (MwoA-DI) and HCs. As the structural change of cortical volume is a long-term process, we combined the MwoA-DI and MwoA-DA as one group to compare it with the control group. Our objective was to test the relationship between the structure and function in MwoA and to explore the pathophysiology of migraine.

## 2. Materials and Methods

### 2.1. Subjects

44 right-handed patients with migraine without aura, including 34 MwoA-DI and 10 MwoA-DA, were recruited from the Department of Neurology at The First Affiliated Hospital of Zhejiang Chinese Medical University. All patients were diagnosed by a neurologist with 10 years' experience according to the International Classification of Headache Disorders, 3rd edition (beta version) [9]. All patients' disease duration attack frequency, VAS, MIDAS, and HIT-6 were recorded. 32 healthy controls were also recruited in this study by Internet and local school advertisements. All participant signed informed consent form. The study was approved by the Ethics Committee of the First Affiliated Hospital of Zhejiang Chinese Medical University.

### 2.2. Image Acquisition

All scans were performed with a 3.0 Tesla MR scanner (GE Discovery MR750 scanner) with an 8-channel head coil to obtain T1-weighted structural images and echo-planar T2^*∗*^-weighted images (EPI). Structural images were obtained by three-dimensional T1-weighted fast spoiled gradient recalled echo (3D T1-FSPGR) sequence: TR = 8.2 ms, TE = 3.2 ms, flip angle = 12, FOV = 256 mm × 256 mm, matrix = 256 × 256, and slice thickness = 1 mm. 180 time points of functional resting state data were acquired by EPI session: TR = 2000 ms, TE = 35 ms, flip angle = 90, slice thickness = 4 mm, slice gap = 1 mm, FOV = 256 mm × 256 mm, and matrix = 64 × 64. All subjects were required to stay awake and instructed to keep their eyes closed during the scan. All the MwoA-DA patients were scanned before they take any painkiller.

### 2.3. Data Processing

All data processing methods have been mentioned in the previous study [10].

#### 2.3.1. VBM Data Analysis

VBM analysis was carried out using Statistical Parametric Mapping (SPM12) (Wellcome Department of Cognitive Neurology, University College London, UK, https://www.fil.ion.ucl.ac.uk/spm), on MATLAB R2012a (MathWorks, Inc., Natick, Massachusetts). All T1 images were normalized to MNI space and segmented into different compartments (white matter, gray matter, and cerebrospinal fluid) using high-dimensional diffeomorphic anatomical registration through the exponentiated Lie algebra (DARTEL) algorithm. All parameters were set according to standard options. Finally, images were smoothed with an 8 mm FWHM smoothing kernel. The normalized, modulated gray matter maps were used for further statistical analysis.

#### 2.3.2. Resting-State Functional Connectivity (rs-FC) Analysis

rs-FC analysis was performed by applying a seed-based approach using the CONN toolbox v15.g (https://www.nitrc.org/projects/conn). The preprocessing steps included realignment, coregistration of each subject's functional and structural images, normalization, and smoothing with an 8 mm full width at half maximum (FWHM) kernel. In addition to these steps, segmentation of GM, white matter, and cerebrospinal fluid (CSF) areas were conducted to remove temporal confounding factors. 24 band-pass filtering was performed with a frequency window of 0.008–0.9 Hz.

To eliminate correlations caused by head motion and artifacts, we identified outlier time points in the motion parameters and global signal intensity using ART (https://www.nitrc.org/projects/artifact_detect). For each subject, we treated images as outliers if composite movement from a preceding image exceeded 0.5 mm or if the global mean intensity was N3 SDs from the mean image intensity for the entire resting scan. Outliers were included in the regression for the first-level general linear model along with motion parameters. Six subjects in the MwoA-DI group, 5 subjects in the MwoA-DA group, and 5 subjects in the HC group showed outlier volumes. In the MwoA-DI group, the mean outlier volume was 6.33 ± 2.50; in the MwoA-DA group, the mean outlier volume was 10.8 ± 2.86; and in the HC group, the mean outlier volume was 6.60 ± 3.05.

The observed clusters from the VBM analysis were extracted as regions of interest (ROIs) for functional connectivity analysis. As in our previous study, first-level correlation maps were produced by extracting the average BOLD time course from each seed and computing Pearson's correlation coefficients describing the correlations between the selected time course and the time courses of all other voxels in the brain. The correlation coefficients were Fisher transformed into “Z” scores, which increased normality and allowed for improved second-level general linear model analyses.

### 2.4. Statistical Analysis

To explore the differences between patients and healthy controls, two sample *t*-tests were performed on the normalized GM and FC maps. A threshold of voxel-wise *p* < 0.001 uncorrected and cluster-level *p* < 0.05 family wise error (FWE) correction was applied to all MRI data analyses. The z-score of the cluster of multiple analyses was extracted to make correlations between patient's clinical data. The demographic and clinical data and correlation analysis were analyzed using the SPSS 25.0 software.

## 3. Results

### 3.1. Clinical Characteristics

A total of 44 migraine patients, including 34 MwoA-DI and 10 MwoA-DA, and 32 controls participated in this study. The VBM analysis covers all participants. One MwoA-DI, one MwoA-DA, and one HC were excluded from rs-FC analysis due to poor registration (Table 1).

### 3.2. VBM Results

**Figure 1:**
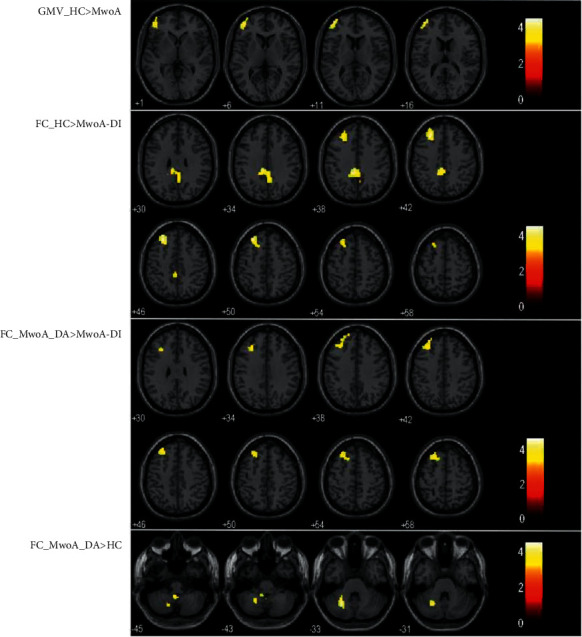
VBM results showed decreased gray matter volume in the left middle frontal cortex (lower) in MwoA patients compared with HCs. rs-FC analysis showed the middle frontal cortex (lower) had a decreased functional connectivity between the middle frontal cortex (upper) and the posterior cingulate cortex in MwoA-DI patients compared with HCs. The middle frontal cortex (lower) had a decreased functional connectivity between the middle frontal cortex (upper) in MwoA-DI patients compare with MwoA-DA patients. The middle frontal cortex (lower) had an increased functional connectivity with left cerebellar lobule VI in MwoA-DA patients compared with HCs.

Compared with HCs, MwoA patients contain much less GM volume in the left middle frontal cortex (Table 2; Figure 1). No area of increased GM volume was found in patients compared to controls at the same threshold.

### 3.3. FC Results

**Figure 2:**
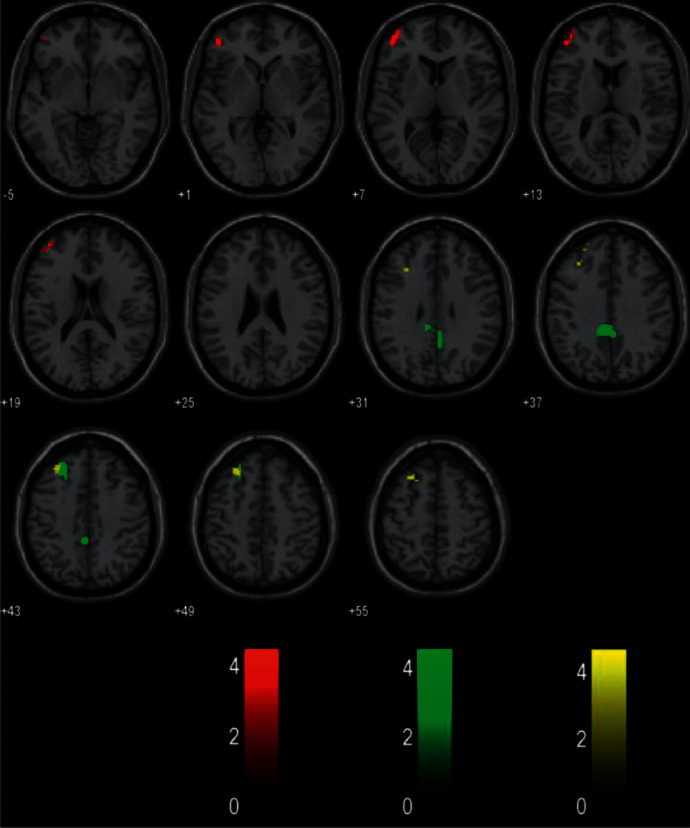
The red color represents the VBM results, the green color represents the rs-FC result of the MwoA-DI group vs. the HC group, and the yellow color represents the rs-FC result of the MwoA-DI group vs. the MwoA-DA group. The rs-FC result found the functional connectivity with the middle frontal cortex (upper) was decreased in MwoA-DI patients compared to HCs and MwoA-DA patients.

To investigate functional networks, the left middle frontal cortex (lower), which represented the ROI, was used to conduct a seed-based analysis. In MwoA-DI patients, compared with HCs, the ROI demonstrated significantly decreased functional connectivity with the left middle frontal (upper), superior frontal, left posterior cingulate cortexes, and the right precuneus. In MwoA-DA patients compared with HCs, the ROI demonstrated significantly increased functional connectivity with the left cerebellar lobule VI. In MwoA-DI patients compared with MwoA-DA, the ROI demonstrated significantly decreased functional connectivity with the left middle frontal (upper) and superior frontal cortex (Table 2; Figure 1). The decreased functional connectivity region of the middle frontal cortex (upper) in MwoA-DI vs. HCs and MwoA-DI vs. MwoA-DA overlapped is shown in Figure 2.

### 3.4. Correlation Results

**Figure 3:**
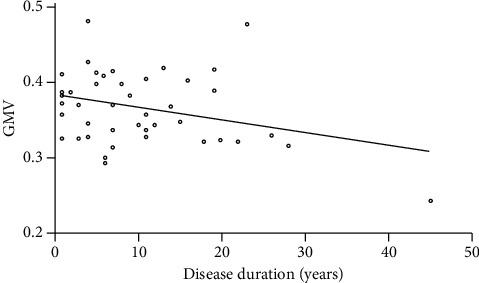
The decreased gray matter volume of the middle frontal cortex in MwoA was negatively correlated with patients' disease duration (*p*=0.040).

**Figure 4:**
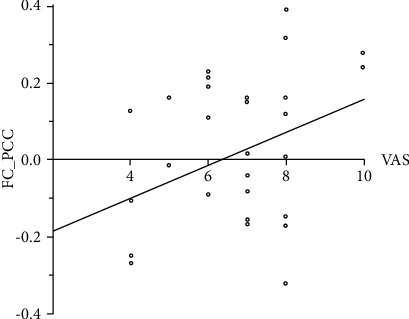
The decreased functional connectivity between the left middle frontal cortex and posterior cingulate cortex in MwoA-DI patients compared with HCs was positively associated with MwoA-DI patients' VAS (*p*=0.023).

The GMV z-score of the surviving cluster was negatively correlated with patients' disease duration (*p*=0.040) (Figure 3). There were no significant correlations between the GMV cluster and MIDAS score, VAS, HIT-6, or attack frequency. The functional connectivity between left middle frontal cortex and posterior cingulate cortex was positively associated with MwoA-DI patients' VAS (*p*=0.023) (Figure 4). There were no significant correlations between other FC clusters and patients' clinical data.

## 4. Discussion

In this study, a generalized decrease in GM volume was observed in MwoA patients in the left middle frontal cortex (lower), and it was correlated with patients' disease duration. The rs-FC analysis revealed that the left middle frontal cortex (lower) in both the MwoA-DA and the HC groups demonstrated significantly increased functional connectivity with the left middle frontal cortex (upper) and left superior frontal cortex compared with MwoA-DI. Also, the left middle frontal cortex (lower) in the MwoA-DI group also showed decreased functional connectivity with the left posterior cingulate cortex and the right precuneus cortex compared with the HC group. This result was positively associated with MwoA-DI patients' VAS. The left middle frontal cortex in the MwoA-DA group demonstrated significantly increased functional connectivity with left cerebellum lobule VI compare with the HC group.

Our study found that the GM volume of the left middle frontal cortex was decreased in migraine patients. From a functional standpoint, the middle frontal cortex is known to serve the role of cognitive evaluation and pain modulation [11]. Previous studies also showed the MwoA patients had decreased GMV in left middle frontal cortex [11, 12]. In this regard, it is suggested that alterations in the function or structure of the frontal gyrus may predispose a patient to pain hypersensitivity from repetitive overstimulation in pain-related processes [13]. Our study also showed that patients with a long duration of disease may have less gray matter volume. Previous studies have demonstrated that the thickness or volume of the left middle frontal cortex is correlated with the attack frequency and the disease duration [14].

We also found decreased functional connectivity between the middle frontal cortex (lower) and the posterior cingulate cortex in MwoA-DI compared with HCs. Previous studies also found dysfunction of the PCC in MwoA [15]. The posterior cingulate cortex is known as the core part of the default mode network and serves a social/emotional function. Some studies have thought that the DMN, including the PCC, may involve in the pain inhibition process and its efficiency [16, 17]. In this study, the posterior cingulate cortex showed a significantly positive correlation with the VAS. Previous studies have shown that the cingulate gyrus may accumulate damage due to the repeated occurrence of migraines [13]. This theory may explain the decreased functional connectivity between the two clusters. As such, the more severe the symptoms are, the greater the disruption in functional connectivity is. However, we did not find any decreased functional connectivity between the PCC and the middle frontal cortex in MwoA-DA patients. This result demonstrated that the balance of DMN coupling between the middle frontal cortex may play a role in pain inhibition. The alteration in PCC functional connectivity in MwoA-DA patients may be due to the compensatory mechanism.

Herein, the functional connectivity was found to be increased between the 6^th^ cerebellar lobule and the left middle frontal cortex in MwoA-DA compared with HCs. Lobule VI of the cerebellum was suggested to serve as an integration center for information across multiple modalities [18]. Researchers in the literature have documented that the cerebellum is associated with the pathophysiological aspects of migraines [19]. Researchers suggested that the function of integration and sensorimotor information at the medulla to cerebellum level may be disrupted [20]. Previous studies have found that the structure of the cerebellum also changes in MwoA patients. Specifically, a higher axial diffusivity, mean diffusivity, and radial diffusivity in the right inferior cerebellum peduncle tract and the spinal trigeminal nucleus were observed [21]. Scholars believe that the descending pain systems between the cerebellum and brainstem play an important role in the development of migraines [22]. Our results showed that the frontal cortex have increased functional connectivity with the cerebellum in MwoA-DA patients. The cerebellum may contact the cerebrum and brainstem to regulate the occurrence and processes of pain during the attack period of migraines.

This study was limited by the small sample size of MwoA-DA patients and the retrospective nature of the research. These limitations result in potential biases. In addition, the MwoA-DA and MwoA-DI patients were not individually matched. Therefore, future studies should explore the specific differences between the attack period and interictal period of migraines to reveal the deep brain mechanisms behind migraines.

## 5. Conclusion

This study demonstrated that the change of gray matter volume was related to altered functional connectivity in MwoA patients. These results suggest the important role of the middle frontal cortex in the pathophysiology of migraines. The PCC, as a part of the DMN, may be involved in pain inhibition. In addition, the cerebellum may serve as a relay center between the cortex and brainstem to regulate somatic pain. Future studies along this direction should be conducted to confirm the findings and explore the deeper mechanisms behind the pathophysiology of migraines.

## Figures and Tables

**Table 1:** Clinical data of MwoA patients and HCs.

	MA			HC
	MwoA-DI	MwoA-DA	Total	
*N*	34	10	44	32
Sex (male)	9	2	11	16
Age (years)	34.44 ± 10.04	36.60 ± 13.02	34.93 ± 10.66	30.63 ± 9.56
Disease duration (years)	10.68 ± 10.03	9.20 ± 3.79	10.34 ± 8.98	—
Attack frequency (times/months)	9.61 ± 8.98	11.90 ± 12.14	10.14 ± 9.68	—
Pain intensity (VAS)	6.94 ± 1.56	7.50 ± 1.35	7.07 ± 1.52	—
MIDAS	33.00 ± 30.22	51.20 ± 48.11	37.14 ± 35.28	—
HIT-6	62.82 ± 6.49	65.90 ± 4.61	63.52 ± 6.20	—

Note: all statistical values used mean ± SD.

**Table 2:** VBM results and functional connectivity results using the VBM cluster as the seed between three groups.

Method	Contrast	Brain region	Cluster size	Peak z-score	MNI coordinates (mm)		
					*x*	*y*	*z*
VBM analysis	HC > MwoA	Left middle frontal cortex (lower)	1301	4.2	−42	57	12
		Left middle orbital frontal cortex		3.94	−48	51	−4.5

FC analysis	HC > MwoA-DI	Left middle frontal cortex (upper)	169	4.21	−27	33	45
		Left superior frontal cortex		3.29	−21	24	60
		Left posterior cingulate	192	4.11	−6	−33	36
		Right precuneus		3.44	6	−54	33
	MwoA-DA > MwoA-DI	Left superior frontal cortex	216	4.16	−30	48	42
		Left middle frontal cortex (upper)		3.86	−24	30	60
	MwoA-DA > HC	Left cerebellar lobule VI	106	4.02	−24	−60	−33

## Data Availability

The datasets used and/or analyzed during the current study are available from the corresponding author upon reasonable request.
